# Network Coordinator Perceptions of Early Childhood Community Systems Building and Development Efforts

**DOI:** 10.3390/children12060802

**Published:** 2025-06-19

**Authors:** Tuyet Mai Ha Hoang, Brandie Bentley, Karen V. Jenkins, Crystal A. Reinhart, Gloria A. Sugg, Karen M. Tabb

**Affiliations:** School of Social Work, University of Illinois at Urbana-Champaign, Urbana, IL 61801, USA; thhoang3@illinois.edu (T.M.H.H.); brandieb@med.umich.edu (B.B.); kvj@illinois.edu (K.V.J.); reinhrt@illinois.edu (C.A.R.); garroy2@illinois.edu (G.A.S.)

**Keywords:** early childhood development, community systems

## Abstract

**Background**: Networks for building and developing community systems to support early childhood rely on the volunteer efforts of organizations and the leadership of coordinators to unite relevant stakeholders. Aims: The purpose of this study was to explore the perspectives of network coordinators from 10 different communities participating in the All Our Kids Early Childhood Networks (AOK Networks) to identify the successes and barriers involved in implementing effective early childhood systems. **Methods**: We conducted two focus group interviews with 10 participants who worked as network coordinators in public health district offices. A semi-structured focus group interview guide was used to examine community coordinators’ perceptions related to lessons learned with community systems development efforts around early childhood outcomes. All interviews were audio recorded and transcribed verbatim. Thematic network analysis was used to analyze all focus group data. **Results**: Three salient themes were identified from interviews with network coordinators: (1) respective strengths of the AOK Networks system building efforts; (2) challenges for coordinators, such as burnout; and (3) the importance of the networks’ role within the community. **Conclusions**: This study contributes to the existing literature by identifying supportive and hindering factors that impact the implementation process to sustain long-term impact of early child community systems building. The findings can be useful for other ongoing government partnerships and community-based programs that use networks and system building strategies across the US.

## 1. Introduction

Building a community-based systems development program to improve early childhood outcomes is a popular approach to support the well-being of very young children and their families and is effective across a variety of global settings [1]. While community systems building is an evidence-supported strategy that has been widely adopted across high-, middle-, and low-income countries [2,3,4], strategies to build community capacity and implementing meaningful change remains a challenging and complex process [5]. Community-level systems change often encounter failures in the areas of implementation and sustained impact [6]. In particular, state-based programs lack the flexibility needed to provide the necessary support for high-quality implementation and sustained community-level efforts, because state agencies are designed to manage and monitor grants and programs (time-limited change), as opposed to supporting the development of infrastructure, systems, and mechanisms for implementation (long-term, sustained change) [5,6].

Although the existing literature provides useful frameworks for guiding systems change in early childhood, there has been limited evaluation of how these frameworks are applied within state-supported, community-based initiatives. The All Our Kids Early Childhood Networks (AOK Networks), a state-supported initiative that promotes community-based systems of care to improve outcomes for young children and their families across one Midwestern state, is informed by both the Active Implementation Frameworks (AIF) and the ABLe Change Framework. AIF emphasizes core implementation drivers including competency, organizational supports, and leadership that are essential for achieving fidelity and sustainability in complex systems [7]. The ABLe Change Framework complements this by focusing on adaptive, equity-centered strategies that promote community readiness, cross-sector collaboration, and co-created solutions [6]. These frameworks inform the AOK Networks’ state-level strategy for supporting locally led systems of care, where network coordinators are key facilitators of implementation in their communities.

The purpose of this study was to explore the perspectives of network coordinators from 10 different communities in the AOK Networks, the most comprehensive and long-standing early childhood systems development initiatives in the state of Illinois. The guiding research question for the study is “*What are the perceived successes and barriers involved in implementing effective early childhood systems in the community network?*”

## 2. Materials and Methods

### 2.1. Setting

AOK Networks was started in 1999 through the collaborative efforts of the Illinois Department of Human Services, Division of Family and Community Services; the Illinois State Board of Education; and local health departments, service agencies, and stakeholders. It comprises state-funded, community-based systems development initiatives that promote healthy pregnancies, well-being of parents/caregivers, and positive development and growth of children from birth to age five. AOK Networks’ mission is to connect partners across diverse service sectors to address local priorities and develop a coordinated system of services for these children and their parents /caregivers. Each county has one AOK Network coordinator, who recruits, maintains, and facilitates interorganizational partnerships across different sectors, as well as engages parents/caregivers and community stakeholders to create a centralized coordinated system of care.

### 2.2. Recruitment and Sample

The research and evaluation team used purposive sampling methods to recruit and enroll 10 focus group participants, one from each of the 10 AOK Networks communities, to ensure representation across all 10 active network sites. These communities are in Illinois counties with diverse geographic characteristics ranging from rural to urban. Eligible participants met two inclusion criteria: (1) fluency in English and (2) currently an AOK Network coordinator. Recruitment was conducted via direct email invitations and follow-up calls. While the sample size was limited to one coordinator per site, it was intentionally designed to capture the unique perspectives of each network’s designated lead, aligning with the study’s focus on community coordinators who lead the implementation of a state-funded, community-based systems development program. After recruiting from 10 sites, we had full participation from all the networks, resulting in a sample of all coordinators in the state of Illinois. Participant characteristics are included in Table 1.

### 2.3. Data Collection

The team conducted two in-person focus group interviews with the 10 participants, using a semi-structured interview guide (Table S2). Three members of the research team were trained focus group facilitators. The focus group interviews were conducted in English. Each focus group lasted 30–60 min, with an average time of 46 min. A sample question is, “How is the AOK Network perceived in your community?” Follow-up questions were asked as needed to gain clarity and a deeper understanding of the participants’ experiences. All participants provided written and signed informed consent and permission for the focus groups to be audio recorded. When possible, an individual note-taker was also present during the focus group sessions to document the discussion. Participants were reminded that they could end their participation at any time without penalty. All study procedures were approved by the University of Illinois Institutional Review Board.

### 2.4. Data Analysis

The team used thematic network analysis to analyze all focus group data. This approach provides a structured process for identifying themes at various levels and offers a visual framework to guide the organization and interpretation of identified themes [8]. To begin the coding process, a trained research assistant transcribed the audio recordings and accompanying field notes verbatim to ensure an accurate and comprehensive record of the focus group discussions. The transcripts were then de-identified and uploaded into qualitative analysis NVivo software for systematic coding and analysis.

Next, all team members independently reviewed each transcript to develop initial codes. The team employed both deductive and inductive approaches during the coding process. Deductive codes were applied based on predefined categories aligned with the research questions, such as implementation strengths and challenges, and the coordinators’ perceptions of their individual and network’s perceived impact within the community. The team also identified inductive codes by uncovering new patterns and consistent ideas that emerged from the data.

**Figure 1:**
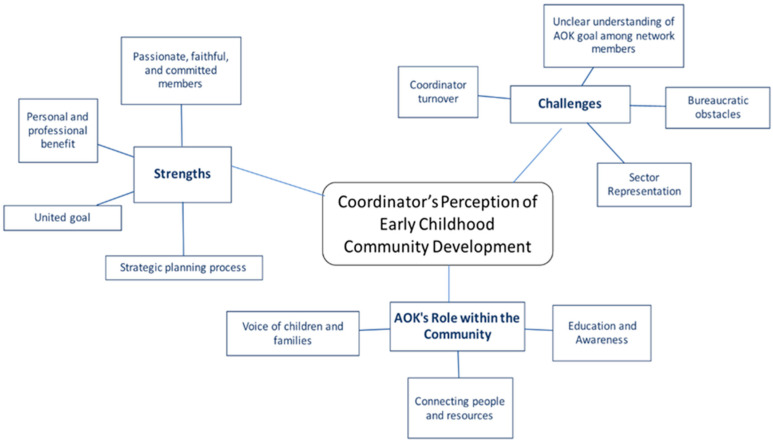
Thematic network analysis.

After the initial individual coding process, the team met to collaboratively organize the basic codes into potential categorical themes. During the interrater coding and discussion phase, we grouped related codes into broader conceptual categories. Team members independently highlighted themes across all transcripts. We also used analytic memos to document reflections and insights as we reviewed each transcript. The team then met weekly to combine notes, identify common themes across the focus groups, and discuss any discrepancies, which allowed for the refinement of themes and subthemes, and enhanced intercoder reliability throughout the process. Finally, each coder individually created a graphic depiction of the thematic network analysis to illustrate the relationships between the identified themes and subthemes. Individual theme maps were compared across coder to reach consensus regarding the final thematic structure. Figure 1 presents results of the final thematic network analysis.

## 3. Results

The results revealed three key themes: (1) respective strengths of the AOK Networks system building efforts; (2) challenges for coordinators; and (3) the importance of the networks’ role within the community. Subthemes addressed topics such as the value of the strategic planning process, obstacles including coordinator turnover and bureaucratic barriers, and the AOK Networks’ role in providing education, awareness, support, and advocacy for children and families. A full list of the themes and accompanying illustrative quotes is provided in Table S1, followed by a summary of the major findings.

### 3.1. Strengths

The first theme the team identified was the strength of the AOK Networks. The benefits gained from AOK Network implementation and participation extended beyond the professional environment, as the participants wholeheartedly believed in the network’s capacity to effect systems-level change as well as create real impact within their local neighborhood and broader community. AOK Network coordinators described their network members’ passion for their work and praised their dedication to achieving the shared goal, a significant strength noted across many counties. Having a shared agenda kept all AOK Networks united under a common purpose and also provided a sense of direction for new and existing members by identifying unique county-level initiatives and goals.

### 3.2. Passionate, Faithful, and Committed Members

Considering the strengths of AOK Network participation and the implementation process, the coordinators all shared the passion and commitment of their AOK Network members, who represented cross-sector organizations, community organizers, parents, and families. One coordinator stated:

“[My network is] passionate about helping families and they want our community to be the best place for families to continue to grow. They see the potential of what we can do together, it keeps them coming back.”

### 3.3. United Goal

In addition, the coordinators discussed how their network members have a good understanding of AOK Network’s mission and all work together toward the overarching goal to improve early childhood outcomes. One said:

“When I go out to meet people to tell them about the network… I think they like to share with each other what’s going on in their organization, but also their ultimate goal is children and families and that’s everybody’s goal, so we’re working towards that together.”

### 3.4. Personal and Professional Benefit

Many of the AOK Network coordinators commented on the personal benefit and meaning that they found in their work. In particular, they discussed how their AOK Network professional duties aligned with their personal interests and values, and how it was especially meaningful to them to observe the immediate impact of their work. For example, one coordinator said:

“So, I like to be very informative in [my work], just to be ready and working with clients; it’s always good to be resourceful that way. I love what I do, and I love visiting my network and getting together with them and coming up with projects together and all that. It’s really cool.”

The coordinators also identified professional benefits, such as a steep learning curve on the job, where multiple skill sets were required compared to other types of community jobs. The coordinators mentioned experiencing a “sink or swim” mentality, but also discussed how much they had learned, ranging from the strategic planning process to relationship building with local organizations and stakeholders. One coordinator mentioned:

“I found the skill wheel and there’s like 50 parts of that wheel and I was like, that’s what it takes to be an AOK Network coordinator, and I’ve used that as a tool to say you know what, I’m not so good with this data analytical piece, that’s somewhere where I really need to grow. Interpersonal skills got it. I need to learn more.”

### 3.5. Real Community-Level Impact

All of the coordinators described their work as meaningful and “making community impact.” Some stated that this was the reason they had applied to be AOK Network coordinators, because they could work directly with community members and organizations to create real system-level impact and immediate change. One coordinator stated:

“I’m in this because of the community and just helping out one family after the other, because you know everything weighs on help and some people need it more than others.”

Another coordinator followed up with a comment about community-level impact:

“Our collective impact work with getting every single child care center in our county to utilize the same curriculum, same assessment process, and now having the school district using the same release so that we can get all of that information from the school about what kids participated in what programs, to look at their reading scores aside that and see are we really having an effect on these specific children’s reading scores by what we’re doing with this project. I’m super proud of that.”

### 3.6. Strategic Planning Process

Although the coordinators discussed difficulties in data collection, assessment, and report generation in the strategic planning process, they also mentioned that going through this process helped them to learn more about applying the framework from ideation to execution and implementation. One coordinator stated:

“[The strategic planning process] allows us to show what we’ve accomplished. Here’s where we started [with] an idea in our heads and put it on paper, but we actually were able to do it, and this is the reason why we did it, because we have a good planning process.”

Another coordinator discussed how the strategic plan provided a good resource for their network members and community:

“I like the whole planning process AOK has. Some people don’t like the strategic plan, but I like everything that there is to it. ABLe is a good framework at times, so it’s a good resource. Just being able to translate that material to the network can sometimes be difficult, but at the same time I feel like I’m educating them, they’re learning how the process is going.”

### 3.7. Challenges

The second theme focused on the challenges of the AOK Network coordinators’ role in the implementation process. Among common challenges, the coordinators shared some practical obstacles stemming from coordinator turnover and burnout, including a continued need for training, technical support, and organizational assistance. The coordinators also discussed the challenges associated with following bureaucratic procedures at both the local and state levels, which hindered their community work at times. Lastly, some coordinators shared difficulty with effectively recruiting, managing, and engaging diverse stakeholders and organizations within their networks.

### 3.8. Coordinator Turnover and Burnout

Consistently across all of the AOK Network counties, the coordinators described the problem of quick turnover in their jobs. This sometimes led to a lack of institutional knowledge. One coordinator mentioned that they had received little information from their predecessor, so they often had to reinvent the wheel:

“In my community, because of the turnaround of the different coordinators, I just came about this year, I really had to build up the network from scratch.”

Burnout was another problem for the AOK Network coordinators because of the demands of managing both local and state priorities in their network members meetings. One coordinator stated:

“I think that is why the last coordinator left, because she catered her meetings to what makes made her members happy and then she tried to do all the work by herself and she burnt herself out.”

### 3.9. Bureaucratic Obstacles

The coordinators described their perceptions of how the strategic planning process was a strength, yet the steps needed to complete assessment and reporting often presented bureaucratic obstacles. One coordinator described their challenges related to the process of reporting and completing compliance documentation:

“So, out of the five days in a week, three of them were on paperwork and whatnot, pulling me out of the community.… [My community partners] are feeling the state is pulling us instead… [of the pull] coming from the local community.”

Additional comments indicated that bureaucratic barriers happen when state-level priorities increase the coordinators’ burden of work when they are implemented at the local level. One coordinator stated:

“Like [using the] AOK Connect [reporting tool] and the bureaucratic way that the assessment process took place and then here there’s more bureaucratic layers at the [local] health department.… It’s harder to do the same job because there are so many more layers that don’t necessarily feel like they’re beneficial.”

These burdens are not unlike similar coordination positions but presented perceived challenges to the coordinators’ ability to spend ample time out in community settings.

### 3.10. Network Recruitment, Management, and Engagement

Another major challenge for coordinators were issues related to network recruitment, management, and engagement. Within this theme, the coordinators talked about a consistent need to increase representation of diverse cross-sector organizations and parents’ voices. As one coordinator said:

“There are always things we can do to improve. There’s always partners still missing from the table that we need to get engaged. There are always opportunities to gain more community support. We definitely need more parental input into the programs and services; I think that’s a statewide issue. So, I think that’s something that we definitely could work toward.”

Although the coordinators recognized the need for recruitment, they also described the difficulties of managing their current networks and engaging their current members in a way that was beneficial for the AOK Network initiatives while maintaining interest from their cross-sector partners. This finding highlighted the challenges of coordinators’ job in balancing different parties’ interests and trying to improve local collaboration for successful implementation of state-level initiatives. A coordinator described this challenge as follows:

“My [AOK Network] members straight up last month told me that they just come for guest speakers and were not happy that was not going to be on the agenda every month and that we were actually going to work towards our initiatives. We’re currently working on restructuring our meetings and outcomes and working really hard.”

### 3.11. AOK Network’s Role Within the Community

The last theme was coordinators’ perceptions of AOK Network’s role within their communities. Participants described AOK as the “voice of children and families.” Through its efforts, AOK Networks connect people to resources and promotes the importance of establishing positive health outcomes during early childhood.

### 3.12. Identifying Needs and Connecting People and Resources

All the coordinators agreed that AOK Network’s role was to identify needs and connect people and resources at the community-level to address issues related to early childhood development. For example, one coordinator said, “If we can’t get things done, we’re going to connect you to the person who can... AOK has the answers.” This theme was consistent across all counties. Another coordinator provided an additional explanation:

“[AOK’s role is] to determine what the areas of need are and then plan around those areas of need, determine how we’re going to either add more services, or change policies, or mindsets, or whatever it is in the community to make whatever changes are needed so whatever that issue happens to be gets addressed.”

### 3.13. Education and Awareness

Another important role for AOK Networks was to bring information and awareness about state and local issues to their community organizations and members. This is important for communities where access to accurate information is difficult. One coordinator stated:

“We do a lot of conversations around the local priorities or issues that are happening in the area and also with AOK we’re able to bring information on the state level so they’re not just talking about what’s happening here locally, but we’re able to bring in that full picture, which I think other networks or early childhood groups in our area really don’t always have that information.”

### 3.14. Voice of Children and Families

Lastly, all the AOK Network coordinators agreed that AOK Networks were seen as an advocate for the youngest, most vulnerable members of their communities. One coordinator said:

“I think in the human service field AOK is perceived as a voice [for] families with young kids.”

The coordinators often stated that they were drawn to this work because they were passionate about improving early childhood outcomes and the well-being of families and young children. For example, one coordinator said: “I feel like in our community, I would say also that we’re seen as a collaborative group. That we’re passionate about representing kids and families.”

## 4. Discussion

This study came together as one part of a multi-part project to evaluate the process of facilitating and sustaining the AOK Networks. We conducted focus groups with AOK Network coordinators and asked them to provide summative evaluations of the strengths and challenges encountered during the implementation process aimed at achieving community- and system-level changes aligned with the AOK Networks’ objectives. Community-based systems development programs based on the collaboration of cross-sector organizations is an evidence-based approach to tackling important public health issues, such as enhancing early childhood outcomes [1,9]. Since 1999, the AOK Networks in Illinois has included counties where coordinators work with local public health districts to establish cross-sector provider networks that support community engagement, family support, and system building to improve early childhood outcomes in their local area. However, state-based programs often face implementation challenges in maintaining long-term impact and system-level changes [5]. Furthermore, succeeding in systems development requires extensive state-to-local support, and coordinators play a key role in the success of the local AOK Networks.

This study explored the AOK Network community coordinators’ perceptions of state-funded community-based programs for early childhood development in maintaining long-term impact. We asked the coordinators to evaluate both their individual roles and the broader AOK Networks’ role in supporting the health and well-being of young children and their families within their respective communities. This is one of the few studies in the US [10] that focuses on the perspectives of community coordinators leading the implementation of a government supported and community-based systems development program. The findings are potentially meaningful for other ongoing state-supported community-based programs across the US and contribute to a deeper understanding of the real-world challenges involved with implementing sustained community- and system-level changes. Overall, the AOK coordinators indicated strong positive support for AOK Networks’ role within their communities. Similarly to other evaluation studies in early child development, the community coordinators indicated both strengths and challenges in the process of implementing local- and system-level changes [11]. One implication of this study is to explore how to better support coordinators in their critical role.

Evaluating the AOK initiative from the perspectives of network coordinators offers a chance to comprehend the real-world strengths and challenges of implementing early childhood systems of care. Effective and sustained implementation often depends on interorganizational partnerships, which are crucial for addressing complex public health issues [10,12]. In the context of community-based systems development, coordinators connect stakeholders, foster collaboration, and strive to enhance access to services for families. Their insights are vital for understanding what supports or limits long-term impact in early childhood community systems.

Early childhood is a significant period in human development where protective factors are established to extend across the life course. The AOK Network coordinators described the importance of AOK Networks’ role within their communities as “a voice of families with young kids” that advocates for resources and support services as well as bridges knowledge gaps between local and state priorities. The coordinators emphasized the importance of AOK Network activities on identifying needs in their communities, committing to strategic planning around those needs, centralizing resources, and connecting cross-sector providers. With the goal of ensuring healthy and positive outcomes for very young children and their families, AOK Networks also provides infrastructure to increase access to services and caregivers’ awareness of local and state-level early childhood support. The coordinators’ responses indicate the importance of state-funded community-based programs in building state-to-local support and maintaining cross-sector provider networks to improve early childhood outcomes.

The AOK Network coordinators highlighted multiple strengths of being a part of the AOK Networks and its implementation, including their passionate and committed cross-sector partners and organizations, who stand with the AOK Networks in working toward its goal. Regarding the process of implementing state initiatives, AOK Network coordinators described it as meaningful for their professional and personal development, where they learned a great deal through the strategic planning and maintaining of cross-sector provider networks. The coordinators discussed the personal satisfaction they felt in seeing community-level changes and real-life impacts that directly resulted from their work within AOK Network communities. The experiences of the coordinators reflect the amount of dedication and personal motivation needed for AOK Networks to work, as the coordinators all recognized how difficult their role was and the demanding skill set that they needed to succeed. The contribution of community coordinators is crucial for state-funded programs such as AOK Networks because of the level of facilitation, engagement, and development of state-to-local structures and local-to-local connections to ensure sustained community-level impact. These findings reflect the principles embedded in both the Active Implementation Frameworks and the ABLe Change Framework, which emphasize the importance of local leadership, strategic alignment, and collaborative infrastructure in sustaining effective systems of care [6,7].

AOK coordinators play an important role in the success and long-term impact of state-funded programs, and the challenges that come with this role are evident in the coordinators’ experiences. The coordinators described three main areas where challenges arose: network management, bureaucratic obstacles, and turnover/burnout. Some of the difficulties related to network management included recruiting and increasing representation of different cross-sector agencies. This process required additional community-level connections and relationship building on the coordinator’s part while also trying to maintain the engagement of existing partners. Coordinators also discussed the challenge of balancing state-level priorities with the interests/buy-in of local community organizations toward a cohesive overarching goal. A similar challenge has been discussed in another evaluation study of community-based projects to address interpersonal violence in Canada [9]. There were gaps in terms of needs and urgency between state and local priorities and the coordinators’ job was to be a voice bridging those differences. The coordinators discussed that this process could be very difficult, especially when they had to follow the many bureaucratic steps required by state-prioritized outcomes (such as in the strategic planning process), while establishing and maintaining a local infrastructure to address immediate needs in their community. Some coordinators mentioned the issue of burnout and high turnover because AOK work required a large skill set and a level of commitment that many coordinators struggled to maintain.

To implement and maintain impact in early childhood systems development requires extensive state-to-local support, and state oversight can help to ensure quality and consistency across sites. This not only includes the support the state provides to the local AOK Networks but also considers the potential obstacles in the implementation process and begins to define the long-term goal of AOK rather than time-limited objectives. Building state-level implementation support for AOK coordinators helps to promote effective partnering with local communities for sustained impact. Coordinators play a key role in the success of the local AOK Networks, so support for their work with special attention to unique challenges in each network may address the issue of burnout and turnover. Therefore, more fully understanding this role and what each local coordinator brings to it is essential to understanding the success of the local networks. We recommend developing a project coordinator questionnaire. The project coordinator questionnaire would assess each coordinator’s skills, background, work style, perceived roles, perceived challenges, and accomplishments. This information would then be linked to other data sources so that we can understand the relationship between what the coordinator does, how the state can support the coordinator, the overall effectiveness of the local network, and the program’s outcomes.

Successful implementation and sustained impact of early childhood systems development requires a significant amount of state-to-local support. Providing ongoing, differentiated technical assistance, training, and support to local AOK Networks and their coordinators is important for long-term success. Topics may include strategic planning, using data for program improvement at the state and local levels, data collection approaches, recruitment, and engagement strategies with local organizations. Technical assistance for state AOK coordinators might address how to use local AOK data and reports to understand the training and technical assistance needs of coordinators, collect data on health trends, and ascertain the effectiveness of any implemented local initiatives, which can be used to advocate for AOK at the state and national levels—that is, to make a case for the value and impact of the AOK Networks. Training and support can also be provided through peer-to-peer networks. Such networks create opportunities for local communities to share success stories and innovations across the state. This may be accomplished through quarterly networking meetings at which such sharing is the primary focus, separate from training.

### Limitations

Our study has several limitations. Although our sample of AOK coordinators were from diverse geographic locations in Illinois, ranging from rural to urban settings, the sample size was relatively small (N = 10). In our case, the sample was fully representative of the entirety of one state. That said, these qualitative findings might be spatially limited and could be different in other states or countries. Furthermore, the evaluation focused on community-based early childhood development programming, so the identified challenges might be unique to the population of children from birth to age five and their families or caregivers. The perspectives of coordination in other age populations may vary. The approach to collaboration across sectors and organizations has potential to be effective to improve early childhood outcomes in both high-income and low-middle-income settings. This study examined perspectives in one high income country, thus more evaluation studies from low- and middle-income countries might be comparative and yield value. Last, we discussed issues related to the implementation and maintenance of early childhood development programs for long-term impact based on the coordinators’ perspectives. The evaluation process should include perspectives from more than just community coordinators and may benefit from the perspectives of state agencies, parents, and families in local communities.

## 5. Conclusions

This study captured the perspectives of community coordinators in the implementation process of a state-funded community-based systems development program. The study contributes to the existing literature by identifying the successes and hindering factors that impact the implementation process to sustain long-term impact. These findings can be useful for other ongoing state-supported community-based programs across the US. In sum, community-based early childhood systems development is an evidence-based approach to address and prioritize the well-being of families and young children.

## Figures and Tables

**Table 1:** Focus group participant demographics from AOK Network coordinators.

Demographic	All Participants *n* = 10	*n* (%)
**Location**		
*County setting*	Rural	5 (50)
	Urban/Suburban	5 (50)
*Race/Ethnicity*	Non-Hispanic White	7 (70)
	Non-Hispanic Non-White	1 (10)
	Hispanic	2 (20)
*Education Level*	Some College/Technical School (13–15 years)	2 (20)
	College Graduate (16 years)	6 (60)
	Graduate School (17 years or more)	2 (20)
*Age*		
	30–39 years	2 (20)
	40–49 years	2 (20)
	50+ years	6 (60)

## Data Availability

Data will be provided upon request. The data are not publicly available due to potential to compromise the privacy of the participants.

## Supplementary Materials

The following supporting information can be downloaded at: https://www.mdpi.com/article/10.3390/children12060802/s1. Table S1: Thematic Network Analysis Results with Illustrative Quotes from Qualitative Focus Groups with AOK Network Coordinators; Table S2: Supplemental Interview Guide Protocol Form.
