# Supplementary material for: Network Coordinator Perceptions of Early Childhood Community Systems Building and Development Efforts
Source: Children (Basel). 2025 Jun 19;12(6):802. doi: 10.3390/children12060802 (PMC12192088; doi:10.3390/children12060802)
Supplement: Supplementary file 1 [file children-12-00802-s001.zip › children-3532423-supplementary.pdf]

Supplemental Table S1. Thematic Network Analysis Results with Illustrative Quotes from Qualitative Focus Groups with AOK Network Coordinators (*N* = 10).

Theme	Sub-themes	Example Quotes
Strengths	Passionate, faithful, and committed members	*"[My network is] passionate about helping families and they want our community to be the best place for families to continue to grow. They see the potential of what we can do together, it keeps them coming back."*
	United goal	*"When I go out to meet people to tell them about the network…I think they like to share with each other what's going on in their organization, but also their ultimate goal is children and families and that's everybody's goal, so we're working towards that together."*
	Personal and professional benefit	*"So, I like to be very informative in [my work], just to be ready and working with clients; it's always good to be resourceful that way. I love what I do and I love visiting my network and getting together with them and coming up with projects together and all that. It's really cool."*
	Real community-level impact	*"Our collective impact work with getting every single child care center in our county to utilize the same curriculum, same assessment process, and now having the school district using the same release so that we can get all of that information from the school about what kids participated in what programs, to look at their reading scores aside that and see are we really having an effect on these specific children's reading scores by what we're doing with this project. I'm super proud of that."*
	Strategic planning process	*"[The strategic planning process] allows us to show what we've accomplished. Here's where we started [with] an idea in our heads and put it on paper, but we actually were able to do it, and this is the reason why we did it, because we have a good planning process."*
Challenges	Coordinator turnover and burnout	*"In my community, because of the turnaround of the different coordinators, I just came about this year, I really had to build up the network from scratch."*
	Bureaucratic obstacles	*"Like AOK Connect and the bureaucratic way that the assessment process took place and then here there's more bureaucratic layers at the [local] health department.… It's harder to do the same job because there are so many more layers that don't necessarily feel like they're beneficial."*
	Network recruitment, management, and engagement	*"My [AOK Network] members straight up last month told me that they just come for guest speakers and were not happy that was not going to be on the agenda every month and that we were actually going to work towards our initiatives. We're currently working on restructuring our meetings and outcomes and working really hard."*
AOK's Role within the Community	Identifying needs, connecting people and resources	*"[AOK's role is] to determine what the areas of need are and then plan around those areas of need, determine how we're going to either add more services, or change policies, or mindsets, or whatever it is in the community to make whatever changes are needed so whatever that issue happens to be gets addressed."*
	Education and awareness	*"We do a lot of conversations around the local priorities or issues that are happening in the area and also with AOK we're able to bring information on the state level so they're not just talking about what's happening here locally, but we're able to bring in that full picture, which I think other networks or early childhood groups in our area really don't always have that information."*
	Voice of children and families	*"I feel like in our community, I would say also that we're seen as a collaborative group. That we're passionate about representing kids and families."*

Supplemental Table S2. Supplemental Interview Guide Protocol Form:

AOK Networks Process Evaluation

Focus Group Interview Guide

Knowledge of the AOK Networks

- In your opinion, what is the role of the AOK Networks?
- In your opinion, what is the goal of the AOK Networks?

Experience with the AOK Networks

- Why did you decide to participate in the AOK Network?
- How effective are the AOK Networks?

Perceived Benefits of the AOK Networks

- How is AOK perceived in your community?
- What are some of the strengths of AOK?
-
- What is AOK doing well?
- What areas of AOK need improvement?
